# What does a failure in anterior cruciate ligament reconstruction really mean? An integrative view

**DOI:** 10.1186/s43019-026-00339-z

**Published:** 2026-07-23

**Authors:** Ruben Dario Arias Pérez, Victor Alfonso Avendaño Arango, German Alejandro Jaramillo Quiceno, Paula Andrea Sarmiento Riveros, Camilo Partezani Helito, João Espregueira-Mendes, Renato Andrade, Margarita Maria Pilonieta Gonzalez

**Affiliations:** 1 Orthopaedic and Trauma Surgery, Pontifical Bolivarian University, Medellín, Colombia; 2 Knee surgery, Salud Sura, Medellín, Colombia; 3 Knee surgery, Clinica del Campestre, Medellín, Colombia; 4 Ortopedia e Traumatologia, Hospital das Clínicas da Faculdade de Medicina da Universidade de São Paulo, São Paulo, Brazil; 5 FIFA Medical Centre of Excellence, Clínica Espregueira, Porto, Portugal; 6 Dom Henrique Research Centre, Porto, Portugal; 7 El Bosque University, Bogotá, Colombia

## Abstract

Anterior cruciate ligament reconstruction (ACLR) is among the most frequently performed and successful procedures in orthopedic surgery. Nevertheless, a substantial proportion of patients experience postoperative “failure,” a term that remains inconsistently defined throughout the literature. This narrative review critically analyzes the heterogeneity of definitions applied to ACLR failure, underscoring persistent gaps, contradictions, and their implications for clinical practice and research. We subsequently propose an integrative definition that aligns with both the clinical and functional objectives of reconstruction. Failure may result from multiple factors, including but not limited to technical, biological, traumatic, and patient-related, and inconsistencies arise when definitions rely exclusively on graft rupture, revision surgery, laxity thresholds, or isolated patient-reported symptoms. Although current consensus statements acknowledge this variability, they fail to provide operational definitions. Consequently, reported failure rates vary considerably, limiting comparability across studies. We advocate defining ACLR failure, as the presence of any of the following: objective or subjective post-operative instability, persistent knee pain, functional limitation including restricted range of motion, documented graft rupture, or new symptomatic meniscal injury in the absence of significant trauma. This multidimensional, patient-centered framework reflects the fundamental goals of ACLR and may serve as a foundation for standardized reporting criteria in future clinical and research endeavors.

## Introduction

Anterior cruciate ligament (ACL) injuries represent one of the most prevalent knee injuries among young athletic populations [1]. The ACL reconstruction (ACLR) is widely considered the standard of care for restoring joint stability and facilitating a return to sport or physical activity [2]. Despite advances in surgical techniques and rehabilitation protocols, a significant proportion of patients experience outcomes that do not achieve the planned surgical objectives. These outcomes are frequently categorized as “failure,” yet no universally accepted definition exists [3].

Definitions of failure vary considerably: some rely on objective criteria such as graft rupture, anterior laxity, or abnormal pivot shift, whereas others emphasize subjective parameters, including perceived instability, pain, or unsatisfactory functional performance [4]. This lack of uniformity complicates comparisons across studies, hinders effective communication among clinicians, and influences reported success rates [5]. Reported success rates after ACLR range from approximately 85–95%, reflecting substantial variability driven by heterogeneous definitions of failure and outcome assessment criteria [6].

Therefore, a critical appraisal of existing definitions is warranted to address these inconsistencies and to propose an integrative, clinically meaningful definition that reflects the fundamental objectives of ACLR: restoring knee stability, optimizing function, alleviating pain, and preventing further joint damage [4, 7]. This narrative review synthesizes the heterogeneity of failure definitions in the literature, evaluates their limitations, and examines the multifactorial mechanisms associated with ACLR failure. On the basis of this analysis, we propose a multidimensional definition that integrates objective measures of mechanical stability, validated patient-reported outcome thresholds, and structural indicators of graft insufficiency or secondary intra-articular damage, aligning outcome assessment with the fundamental goals of ACLR.

## Variability in definitions of ACLR failure

In contemporary literature, the term “failure” of ACLR has been defined in multiple, often incompatible ways. A recent systematic review [3], including more than 100 clinical studies, highlighted the extent of this heterogeneity. Importantly, many studies used more than one criterion to define failure, which explains why the cumulative percentages exceed 100%. Approximately 35% of studies included physical examination findings (such as a positive Lachman or pivot shift test), 37.5% incorporated the need for revision surgery, and 35.6% relied on imaging confirmation of graft rupture through magnetic resonance imaging (MRI) or radiographs. Only about 17% incorporated patient-reported outcome measures (PROMs) or patient-perceived instability into their definitions. Less frequently, some authors required direct arthroscopic visualization of graft failure, an extremely specific but impractical criterion, used in approximately 12% of studies. In rare cases (< 1%), unrelated procedures such as posterior meniscectomy were considered indicators of failure.

In recent years, the concept of the Patient Acceptable Symptom State (PASS) has further illustrated the diversity of outcomes considered relevant in defining failure. PASS thresholds have been established for commonly used PROMs after ACLR. For the International Knee Documentation Committee (IKDC) subjective score, a cohort from the Delaware-Oslo group [8], reported a threshold of 76.2 points (95% CI 72.1–79.4) at 10 years of follow-up, while Müller et al. [9], found a similar PASS value of 75.9 points at one to five years post-surgery. For the Lysholm score, a PASS value of 85 at a minimum of 2 years of follow-up has been proposed [10]. Patients who do not reach these thresholds often report knee instability, pain, or functional limitations despite radiological evidence of a structurally intact graft. Therefore, incorporating PASS criteria (e.g., IKDC ≥ 76 or Lysholm ≥ 85) expands the definition of failure beyond graft rupture or the need for revision to recognize the domain of functional or patient-perceived failure, even when structural success appears preserved.

This wide variability in definitions naturally produces equally disparate reported failure rates. Depending on the criteria adopted, the incidence of ACLR failure varies dramatically [11]. A systematic review [3] demonstrated that failure rates ranged from 0 to 100% across the studies analyzed, underscoring that virtually any reconstruction could be labeled as either “success” or “failure” depending on the parameters chosen. For instance, if failure is defined strictly as the need for surgical revision, only cases involving graft re-rupture or severe instability requiring reoperation are counted as failures. Conversely, if failure is defined using clinical testing such as side-to-side anterior laxity > 5 mm (compared with the contralateral knee) measured by an arthrometer, or persistent pivot shift (of any grade) on clinical testing, a much higher proportion of “failed” knees will be identified, even when patients have an intact graft, remain asymptomatic or have successfully returned to their sport [3–5].

To mitigate this variability, some authors have adopted composite definitions combining multiple parameters. Common examples include defining failure as clinically or imaging confirmed graft tear, persistent knee instability requiring surgical revision, or classification in the lower IKDC objective grades (C or D). Others have incorporated patient-reported instability during daily activities or specific PROM thresholds [8, 12]. Additional inconsistency stems from differences in follow-up duration: early failures are often related to technical errors or traumatic reinjury and occur within the first postoperative year; these differ fundamentally from late failures driven by progressive laxity, degenerative changes, or secondary pathology. Many studies do not specify the time frame used to evaluate failure, further complicating interpretation and comparison of results [13, 14].

Recognizing these discrepancies, a systematic review of randomized controlled trials [5], confirmed substantial heterogeneity among high-level of evidence studies and advocated for the development of a universal, multidimensional definition integrating both objective and subjective outcome measures. Such an approach would more accurately reflect clinical reality and enhance the comparability of research findings across studies. Furthermore, although the ACL Study Group addressed this issue during its 2023 and 2026 meeting; however, no formal consensus has yet been established [15].

### Definitions proposed by scientific societies and key authors

Given the wide variability in how ACLR failure is conceptualized, several expert groups, scientific societies, and multicenter research networks have proposed structured definitions. However, these definitions rely on different conceptual frameworks, ranging from purely structural graft insufficiency to revision-based endpoints or patient-reported functional impairment. Table 1 summarizes representative definitions and categorizes them according to their primary conceptual basis to facilitate comparison and highlight prevailing patterns in the literature.

**Table 1:** Reported definitions of ACLR failure and their primary conceptual framework

Source (Year)	Definition of ACL failure	Primary definition framework
Freddie Fu et al. (1995)	Instability, pain, stiffness, or inability to return to function; reconstruction may be considered failed even if graft is intact [16]	Symptomatic/functional
Noyes et al. (2001)	Graft insufficiency confirmed by ≥ 6 mm side-to-side difference (KT-1000), grade 3 + Lachman/pivot shift, or recurrent functional instability [17]	Objective/structural
MOON Cohort (2007–2018)	Graft re-rupture requiring revision surgery [18–20]	Revision-based
MARS Cohort (2013)	Documented graft insufficiency: rupture on imaging, anterior laxity > 5 mm, abnormal clinical tests, functional instability, or arthroscopic confirmation [21]	Combined structural + objective + symptomatic
Panther Consensus (2020)	Recommends explicit definition in each study; may include need for revision, persistent subjective instability, abnormal laxity (Lachman/pivot shift), or graft disruption on MRI/arthroscopy [22]	Mixed (objective + revision + symptomatic)
Getgood et al. – STABILITY trial (2020)	Composite outcome: need for ACL revision or persistent symptomatic rotatory instability with abnormal pivot shift [23]	Revision-based + objective
ESSKA Consensus (2022)	“Abnormal knee function associated with a previous ACLR.” Includes graft insufficiency with abnormal laxity or inability to achieve a functional knee according to expected outcomes [24]	Combined structural + functional
Diquattro et al. (2023)	History of ACLR with new clinical instability symptoms, positive Lachman and/or pivot shift tests; ideally confirmed by imaging (stress radiographs or MRI) [13]	Combined objective + symptomatic
Sonnery-Cottet et al. (2025)	Clinical instability (positive Lachman and pivot-shift tests) with side-to-side anterior laxity > 3–5 mm (Rolimeter), confirmed by MRI [25]	Structural + objective

*ESSKA*, European Society of Sports Traumatology, Knee Surgery and Arthroscopy; *MARS*, Multicenter ACL Revision Study; *MOON*, Multicenter Orthopaedic Outcomes Network; *MRI*, Magnetic Resonance Imaging; *ACLR*, Anterior Cruciate Ligament Reconstruction

## Causes and factors associated with ACLR failure

**Fig. 1:**
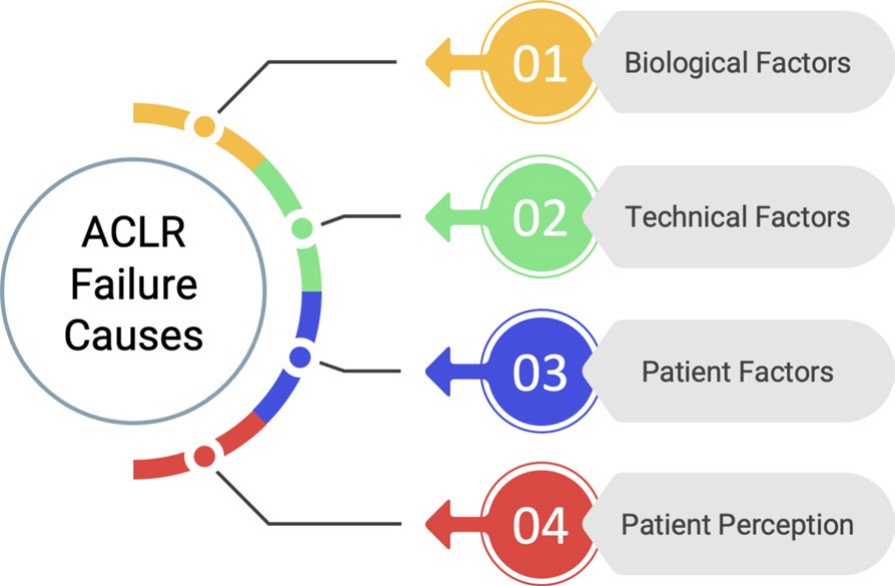
Cause and factors associated with failure of the reconstructed ACL

ACLR failure is usually a multifactorial phenomenon, resulting from the interaction of various factors related to the surgery, the patient’s biology, and the recovery process [7]. As the literature points out, it is often difficult to attribute failure to a single cause. The main categories of factors involved are critically discussed below (Fig. 1).

### Biological factors

Biological factors encompass causes of ACLR failure related to graft healing, incorporation, and the individual biological response to surgery. Although less frequent than technical or traumatic mechanisms, they represent an important subset of failures in which no clear technical error or reinjury can be identified [13]. In the literature, biological failure generally refers to cases where the graft does not integrate adequately or undergoes premature degeneration despite appropriate surgical technique and the absence of a significant traumatic events [13]. A review reported that approximately 8% of failures were attributed exclusively to biological causes [13]. These mechanisms include:

Poor graft integration: in some patients, the graft, whether autograft or allograft, fails to achieve adequate incorporation or biological healing within the bone tunnel [26]. Several biological and patient-related factors may negatively influence graft healing, including smoking, tetrahydrocannabinol (THC) use, systemic conditions such as diabetes or collagen disorders, nutritional deficiencies, and individual variability in healing capacity [27]. While these factors have been associated with impaired graft maturation and delayed incorporation, their direct relationship with clinical graft failure remains less clearly established [28]. Inadequate biological integration may predispose the graft to progressive elongation over time, even in the absence of overt trauma, potentially resulting in increased sagittal or rotational laxity [29–31].

Immunological reaction or graft rejection: although uncommon, allografts can theoretically trigger an immune response that interferes with incorporation. More relevant clinically is the well-documented higher tear rate of allografts compared with autografts in young, active individuals [26]. For instance, adolescent males receiving allografts exhibit significantly higher rates of re-tear and revision than those treated with autografts, underscoring that graft selection, although primarily technical, also carries a biological dimension [32]. Consequently, autografts remain the preferred option in younger athletic populations.

Postoperative infection: infection following ACLR occurs in approximately 1–2% of cases and can significantly compromise graft integrity. Beyond overt septic arthritis, subtle or low-grade infections may manifest as persistent pain or unexplained laxity, or tunnel enlargement [33, 34]. In early failures without an evident mechanical cause, infection, particularly from atypical organisms or fungi, should be considered. Preventive strategies such as vancomycin-soaked autografts, have demonstrated a substantial reduction in postoperative infection rates and are increasingly adopted in clinical practice [35, 36].

Arthrofibrosis: at the opposite end of the biological spectrum, excessive scar tissue formation and arthrofibrosis can also constitute a form of functional failure. Although it does not involve graft laxity, the resulting restriction in the range of motion (typically loss of extension) prevents the knee from achieving the functional goals of ACLR. Arthrofibrosis may be influenced by technical aspects (e.g., anterior femoral tunnel placement causing graft impingement, inadequate early rehabilitation, and arthrogenic muscle inhibition); however, it largely represents an exaggerated biological response to surgery. Severe cases may require manipulation under anesthesia or arthroscopic release, interventions that may be clinically categorized as failure [11, 14].

Other biological factors: additional biological contributors include hormonal or genetically mediated susceptibility to ligament injury [37]. Genetic predisposition has been associated not only with noncontact ACL injuries but also with variations in inflammatory response and extracellular matrix turnover, which may increase the risk of arthrofibrosis following ACLR [38, 39]. Patients with generalized hyperlaxity may exhibit progressive graft stretching despite technically well-performed reconstructions, owing to intrinsic connective tissue characteristics [40]. Hyperextension greater than 5 degrees is already considered a risk factor for graft rupture [41], and cases of hyperextension greater than 6.5 degrees may have up to 14 times greater chances of graft rupture [42]. Chronic synovitis or preexisting osteoarthritis may also alter the intra-articular environment and compromise graft maturation. Although specific biomarkers predictive of failure have not yet been established, ongoing research into synovial cytokines, growth factors, and matrix metalloproteinases aims to clarify their potential role in graft longevity [43].

In summary, biological factors, although often less apparent than technical errors or traumatic mechanisms, represent critical contributors to ACLR failure. These factors frequently overlap with technical aspects such as graft choice and tunnel positioning and should be considered when no clear mechanical cause is identified. Preventive strategies include optimizing graft choice, minimizing infection risk through meticulous surgical technique and prophylaxis, and promoting conditions favorable to graft integration. Nevertheless, a proportion of biological failures remains unavoidable owing to individual variability in graft healing.

### Technical factors

Technical factors refer to intraoperative components of ACLR which, when suboptimal, can compromise graft function and long-term stability. They are consistently regarded as the most common and preventable causes of failure. Historical reports and comprehensive reviews estimate that technical errors account for 22% to 79% of ACLR failures [44]. In a meta-analysis [45] involving 3.567 ACL revision cases, technical errors were identified as one of the leading causes of failure, surpassed only by acute traumatic reinjury. Similarly, malposition of the tunnels, inadequate graft tensioning, and failure to recognize or treat concomitant pathology, particularly meniscal and articular cartilage lesions, are reported as frequent findings in failed ACLRs [26, 46].

**Fig. 2:**
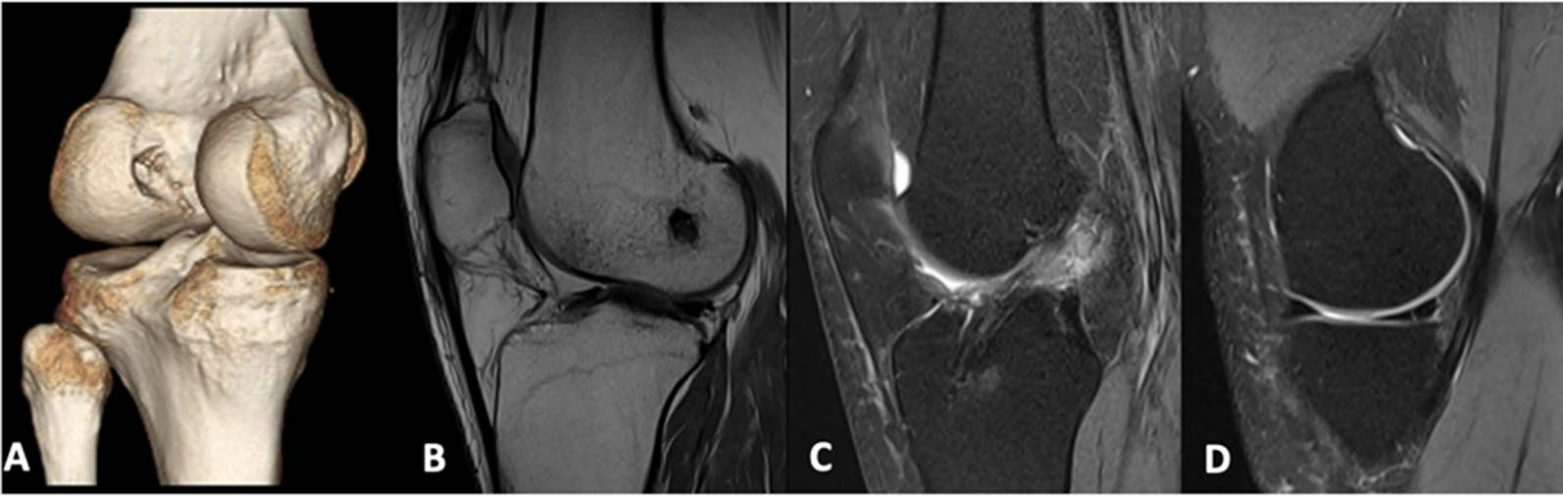
Example clinical case of femoral tunnel malposition. A 34-year-old patient, four years after ACLR, presented with 1 year of instability and intermittent pain, without a new traumatic event. Physical examination revealed positive pivot shift but negative Lachman and anterior drawer tests; medial meniscal tests were positive. **A.** CT 3D reconstruction demonstrated femoral tunnel malposition. **B.** T1 sagittal MRI confirmed tunnel malposition. **C.** Proton Density Fat-Suppressed (PD-FS) sagittal MRI showed a markedly thinned graft. **D.** PD-FS sagittal MRI revealed a medial meniscal tear

Among technical errors, tunnel malposition is by far the most prevalent. Femoral tunnel malposition accounts for approximately 63% of technique-related failures, followed by tibial tunnel malposition (~ 7%), inadequate fixation (~ 2%), and excessive tunnel widening (~ 1%) [13]. A femoral tunnel placed too anteriorly and high (i.e., in a more vertical orientation) fails to replicate the native ACL’s obliquity, thereby compromising rotational control and predisposing the graft to elongation under load, (Fig. 2), [13]. In addition, small graft diameter is another well-documented risk factor: soft-tissue grafts measuring < 8 mm have consistently demonstrated higher rupture rates, making graft size a critical technical consideration [47–49]. In a recent study, by Helito et al. [50] suggested that an associated extra-articular procedure could mitigate the limitations of small diameter ACL grafts; however, this approach requires further validation.

Unrecognized Concomitant Injuries: failure to diagnose and appropriately manage associated knee injuries during the primary ACLR accounts for approximately 15% of graft failures [13, 44]. These injuries may involve the anterolateral ligament, posterolateral corner, medial collateral ligament (MCL), and menisci [26, 44, 51]. Posterior root tears of the lateral meniscus, present in 8–14% of primary ACL injuries, are identified in up to 40% of failed ACLRs and significantly contribute to rotatory instability and anterior tibial translation when left untreated [52, 53]. Ramp lesions of the medial meniscus, occurring in 9–40% of ACL tears, are frequently underdiagnosed and may increase graft loading if not addressed [54]. MCL injuries, observed in 20–38% of ACL ruptures, can also compromise outcomes when inadequately managed [55]. A large cohort study (~ 10,000 patients) reported a 1.5-fold higher risk of ACL revision in nonoperatively treated MCL injuries compared with isolated ACL tears [56].

Lateral extra-articular procedures (LEAPs): adding a LEAP, such as an anterolateral ligament reconstruction or iliotibial band tenodesis, provides clear benefits in high-risk patients [23, 57, 58], not only in primary but also in revision ACL cases [59]. A network meta-analysis of randomized trials [60] found that combining ACLR with anterolateral ligament reconstruction or iliotibial band tenodesis reduces the likelihood re-rupture by approximately 73%. Long-term data from the Scientific Anterior Cruciate Ligament Network International (SANTI) group (~ 8-year follow-up) showed failure rates of 2.7% with isolated ACLR versus 0.7% with LEAP augmentation [61]. Currently, the adoption of LEAPs is considered one of the pillars of ACLR owing to its strong association with reduced failure rates.

### Patient-related factors

Patient-related factors encompass individual characteristics that may predispose to or contribute to ACLR failure, either independently or in combination with technical and biological mechanisms. The most relevant factors described in the literature include the following:

Age and activity level: younger age is one of the most consistently reported risk factors for ACL graft failure. Patients younger than 20–25 years, particularly active adolescents, demonstrate significantly higher re-rupture and revision rates compared with older individuals [27, 62]. National registry data indicate that individuals under 20 years may have up to a threefold higher risk of revision compared with those over 20, and substantially higher risk than patients older than 40 [63]. Each additional year of age appears to slightly reduce the likelihood of re-rupture [64].

This increased risk is largely mediated by higher exposure to pivoting and contact sports, greater mechanical demand on the graft, and premature return to sport before adequate biological maturation and rehabilitation completion [44]. When adjusted for confounding factors (return to level I sports and passing the return to sports criteria), there is no longer an association between age and risk of sustaining a second ACL injury [65].

Sex: the influence of biological sex on ACLR failure remains controversial [44]. Some registry studies report higher revision rates in men, possibly reflecting greater participation in high-risk sports [66, 67], whereas others suggest increased risk in women or no significant differences once activity exposure is controlled [68, 69]. Although hormonal and anatomical factors have been proposed, current evidence suggests that sport type, biomechanical patterns, and activity level are more relevant determinants than sex alone. When exposure and mechanical demand are comparable, re-rupture rates appear to converge between sexes [44].

Individual anatomy: certain anatomical characteristics increase mechanical stress on the ACL graft by promoting anterior tibial translation, rotational instability, or graft impingement. The most consistently reported anatomical risk factors include:

o Increased posterior tibial slope (> 12°: An excessive posterior tibial slope augments anterior tibial shear forces during weight-bearing and dynamic activities, thereby increasing graft load and strain. Slopes greater than 12° have been consistently associated with higher rates of ACL graft failure [70]. In young patients with steeper tibial slope, long-term graft rupture rates may reach 50–70% [14]. Surgical correction of excessive posterior tibial slope through slope-reducing osteotomy has been shown to improve knee stability and may reduce the risk of graft re-rupture [71, 72]
o Excessive genu recurvatum, which may predispose to graft elongation or persistent instability owing to increased anterior shear forces at terminal extension [62].
o Narrow femoral intercondylar notch, potentially leading to graft impingement during extension; in selected cases, notchplasty may be considered to reduce mechanical abrasion [73].
o Coronal malalignment, particularly varus alignment, which increases medial compartment loading and may alter ACL stress distribution, although valgus alignment may also influence joint mechanics [74–77]. In cases of marked malalignment, corrective osteotomy may be necessary to reduce the risk of secondary graft failure.

Rehabilitation compliance: patient adherence to rehabilitation is critical for graft protection and functional recovery. Both insufficient participation in physical therapy and premature or excessive loading can compromise reconstruction outcomes [44]. Failure to regain full extension early increases the risk of stiffness, while inadequate quadriceps and hamstring strengthening impairs dynamic stability and predisposes to reinjury [78]. Conversely, early return to high-risk sports before adequate graft maturation may result in re-rupture. The decision on return to sports must be guided by objective criteria and not only based on time. Although challenging to quantify, suboptimal rehabilitation remains a recognized contributor to ACLR failure [78], and most often athletes fail to achieve established cut-offs of important clinical and functional objective criteria before they return to sports [79]. Educational strategies and appropriate expectation-setting are therefore essential to reduce this risk [13].

Generalized ligamentous hyperlaxity: patients with generalized hyperlaxity (e.g., high Beighton scores, marked joint hyperextension) exhibit greater baseline joint translation and may continue to demonstrate increased laxity even after technically sound ACLR [26, 40]. The European Society of Sports Traumatology, Knee Surgery and Arthroscopy (ESSKA) consensus study identified hyperlaxity as a risk factor for primary injury and potential re-injury [80]. In such patients, tailored surgical strategies, such as reinforced graft constructs or adjunctive LEAPs, may be considered [81], along with careful rehabilitation to avoid excessive graft strain.

Importantly, residual laxity in hyperlax individuals should not automatically be classified as failure. According to the multidimensional framework proposed in this review, increased translation becomes clinically relevant only when it is accompanied by functional instability, patient-reported symptoms, or structural graft compromise [82]. Thus, isolated objective laxity in the absence of symptomatic or functional limitation does not necessarily meet the criteria for ACLR failure.

Return to high-risk sport: consistent with the discussion on age and activity level, patients who return to pivoting or contact sports (soccer, basketball, rugby, skiing, military training) face a substantially increased risk of reinjury to the reconstructed graft or contralateral ACL [27, 83]. Those returning to level I sports after had a ~ fourfold higher reinjury rate [84]. Estimates suggest a 5–10% rate of re-rupture within five years among those who resume their preinjury level of sport [5]. Importantly, failure in these cases often reflects the inherent risk of high-energy pivoting activities rather than surgical shortcomings. As such, analysis of failure must incorporate the concept of “exposure risk,” recognizing that an otherwise successful reconstruction does not confer immunity under demanding athletic conditions [5, 44].

## Subjective and objective evaluation of failure: discrepancies and clinical relevance

A major challenge in defining ACLR failure is the frequent mismatch between objective clinical findings and the patient’s subjective experience. Patients may demonstrate biomechanical insufficiency on examination or imaging while reporting satisfactory function and no perceived instability [5]. Conversely, others exhibit normal clinical stability, intact grafts on MRI, and no measurable laxity, yet describe their knee as unstable, unreliable, or functionally limiting [5]. Helito et al. reported that patients with preoperative knee hyperextension who regained hyperextension after ACLR demonstrated greater objective knee laxity than those who recovered full extension without hyperextension. Nevertheless, recovery of full range of motion was associated with superior subjective outcomes [85].

This divergence highlights the need to assess outcomes from both objective and subjective perspectives. Objective evaluation relies on clinical examination (Lachman and pivot shift), instrumented testing such as KT-1000 arthrometry or Lachmeter, and imaging modalities including MRI or stress radiographs to detect abnormal translation or graft insufficiency. However, mechanical findings do not always correlate with functional limitation. Meta-analyses show that 10–30% of patients exhibit mild residual laxity without perceived instability or activity restriction [86, 87], supporting the concept of a “tolerable laxity threshold.”

Subjective evaluation, by contrast, reflects real-world functional performance. Some patients with structurally intact grafts report subjective feelings of instability, avoidance of pivoting activities, or lack of confidence [44]. These symptoms may result from neuromuscular deficits, proprioceptive impairments, poor psychological readiness, or persistent pain. When they significantly limit daily activities or sports participation, the ACLR may represent a functional failure despite preserved mechanical integrity [3].

This discrepancy has led to growing consensus that no single parameter sufficiently defines ACLR failure. Contemporary frameworks therefore advocate composite definitions integrating structural integrity (e.g., graft rupture or pathological laxity) with functional outcomes. Importantly, validated PASS thresholds operationalize subjective dissatisfaction in a reproducible manner, transforming patient perception into quantifiable criteria applicable to both clinical decision-making and research comparability. Applying established PASS cut-offs for instruments such as the IKDC or Lysholm scores allows subjective failure to be standardized without disregarding the patient-centered dimension of outcome assessment.

In clinical practice and research, this multidimensional reporting approach has significant implications. Best practices recommend simultaneous reporting of graft rupture rates, revision rates, objective laxity measures, and the proportion of patients failing to achieve PASS thresholds. This enables assessment not only of mechanical graft stability but also of whether reconstruction achieves an acceptable symptom state from the patient’s perspective.

In summary, ACLR failure may manifest in two complementary domains: structural failure, reflecting graft tear or biomechanical graft insufficiency; and functional failure, reflecting inability to reach an acceptable symptom state. A clinically meaningful and methodologically robust definition must incorporate both domains.

## Proposal for a new definition of failure in ACLR

From the critical analysis of the literature and the concepts previously discussed, no unidimensional definition, whether purely objective or purely subjective, adequately captures the full spectrum of ACLR failure. A meaningful definition must align with the fundamental clinical objectives of the procedure: (1) restoration of knee stability; (2) recovery of function and full range of motion enabling return to activity; (3) achievement of a pain-free joint; and (4) long-term protection of the knee from secondary damage, particularly meniscal injury.

**Fig. 3:**
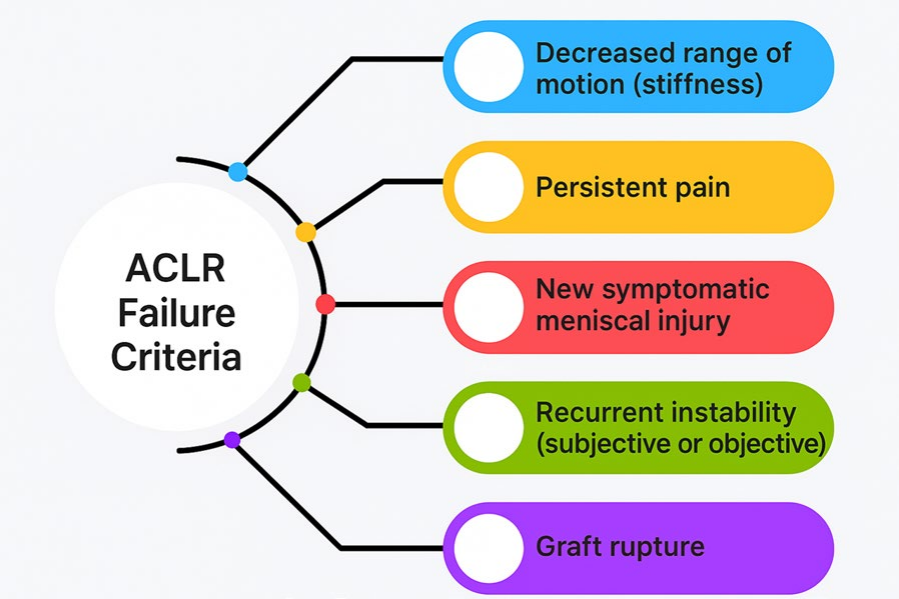
Criteria for defining failure of ACLR

On the basis of these principles, we propose defining failure of ACLR as the presence of any of the following: (1) an unstable knee, whether detected objectively or perceived subjectively by the patient; (2) persistent clinically-relevant pain; (3) functional limitation, including loss of range of motion; (4) documented graft rupture; and (5) new symptomatic meniscal injury in the absence of relevant new trauma. (Fig. 3).

Objective knee instability: objective instability refers to abnormal laxity on clinical or instrumented evaluation that compromises anterior and/or rotational knee control. This includes a clearly positive Lachman test, a moderate-to-severe pivot shift, or increased anterior translation measured with KT-1000/2000 or digital arthrometry beyond accepted normative values [3]. Additionally, stress MRI combined with instrumented testing devices, such as the Porto-Knee device, has demonstrated a strong correlation between global tibial rotation and pivot-shift grading, suggesting high sensitivity for detecting ACL-related multiplanar instability [88, 89]. These findings indicate that the graft is not mechanically fulfilling its stabilizing function. Notably, minor laxity (e.g., Lachman grade 1 or pivot glide) does not necessarily constitute failure unless it affects function; thus, the emphasis remains on clinically relevant instability.

Subjective perception of knee instability: patients may report recurrent episodes of “give-way,” avoidance of pivoting activities, or diminished confidence in the reconstructed knee despite normal objective stability [22]. Subjective functional instability may also manifest during activities that challenge dynamic knee stability and may negatively affect the patient’s ability to return to previous levels of activity. Although patients with instability-related symptoms may demonstrate poor performance on validated tests (e.g., hop tests with poor interlimb symmetry) and may report lower scores on PROMs such as IKDC, Knee injury and Osteoarthritis Outcome Score (KOOS), or Lysholm, these findings should be interpreted as complementary measures of function rather than direct indicators of instability. This acknowledges that instability-related symptoms represent a distinct clinical domain that may contribute to an unsuccessful outcome after ACLR, even in the absence of objective evidence of graft failure.

Persistent pain, stiffness, or other joint-related symptoms originating from ACLR: presence of chronic post-ACLR knee pain or significant loss of range of motion (limited knee flexion or extension owing to arthrofibrosis or scarring) that was not present prior to surgery or is a consequence of the surgical procedure and negatively impacts function. Although ligamentous stability may be intact, patients may continue to experience functional limitations, reduced activity levels, or dissatisfaction with the outcome of surgery. Low scores on PROMs or failure to achieve PASS thresholds may reflect these persistent symptoms and indicate that the reconstruction has not fully achieved its intended patient-centered goals. Therefore, a knee with persistent pain ≥ 5/10 or a mobility restriction of > 5°–10° of flexion or extension that affects gait or activity represents an adverse outcome comparable to failure, given that the goal was a symptom-free and functional knee [16].

Documented graft rupture: this remains one of the most consistently reported and widely accepted indicators of ACLR failure. This finding represents unequivocal structural failure of the graft and is therefore considered a definitive criterion across multiple studies and consensus statements. Graft disruption can be confirmed through MRI, typically demonstrating discontinuity, abnormal signal intensity, or loss of graft integrity, or directly via arthroscopic visualization, which provides the most conclusive assessment [3]. Because graft rupture inherently compromises knee stability and function, its identification is an unequivocal finding to define postoperative failure and guide the need for revision surgery [7, 14].

Progressive structural damage to the knee owing to ligament insufficiency: the occurrence of a new symptomatic meniscal tear or the progression of a previously documented meniscal lesion after ACLR may represent a consequence of persistent biomechanical insufficiency of the reconstructed knee. Given the well-established role of the menisci, as secondary restraints to anterior tibial translation, persistent ligamentous insufficiency may increase mechanical stress across these structures and contribute to secondary meniscal damage [90]. On the basis of these biomechanical principles, we propose that symptomatic meniscal injury following ACLR should be regarded as a potential sign of reconstruction failure. However, we acknowledge that this component of the proposed framework remains theoretical and requires further clinical validation. Nevertheless, this association is frequently observed in clinical practice and may provide valuable information when assessing patients with suspected reconstruction failure.

**Fig. 4:**
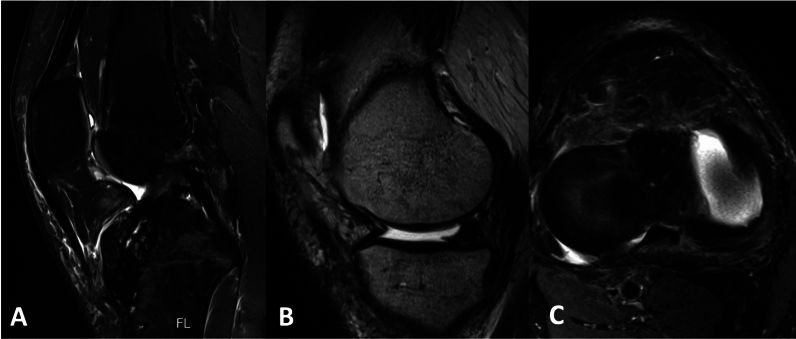
Example of a clinical case of symptomatic meniscal tear. A 37-year-old patient, 8 months after ACL and anterolateral ligament reconstruction with medial meniscus repair, presented with new-onset pain without a new traumatic event. Stability tests were negative. MRI demonstrated: **A.** Properly positioned tunnels with graft in the healing phase and a double-PCL sign; **B.** Medial meniscus tear on T1 sagittal sequence; **C.** Bucket-handle tear on axial Proton Density Fat-Suppressed (PD-FS)

Accordingly, the authors believe that the development of a new symptomatic meniscal lesion is interpreted as a structural consequence of insufficient ligamentous control and reflects failure to achieve the primary objective of reconstruction, restoration of stable knee kinematics and protection of intra-articular structures, (Fig. 4), [13].

## Temporal considerations

Failure may manifest at different time points following ACLR. Early failures (within the first 3–6 months) are typically related to technical factors or acute trauma and often present with clear structural instability. By contrast, intermediate or late failures (≥ 1–2 years) may result from progressive graft elongation, biological insufficiency, or secondary intra-articular damage.

Although the timing and underlying mechanisms may differ, the defining criteria remain consistent across phases. For research purposes, failures may be categorized as early or late to facilitate subgroup analysis, but the multidimensional definition itself is independent of time since surgery.

## Rationale and implications

This proposed framework aligns with contemporary recommendations advocating for composite outcome measures that integrate objective stability and patient-reported outcomes [4, 14]. It recognizes that graft integrity alone may not fully capture the outcome of ACLR and suggests that successful reconstruction should ideally restore knee stability, optimize functional recovery, and prevent secondary intra-articular damage, including symptomatic meniscal injury. By incorporating this preventive dimension, the framework extends beyond traditional rupture-based definitions and reflects the broader long-term objectives of ACLR. Importantly, when studies intend to evaluate structural graft rupture as an isolated endpoint, this outcome should be explicitly described as “graft tear” or “graft rupture” rather than broadly labeled as “failure,” as these terms are not interchangeable and represent distinct conceptual constructs.

By integrating structural, functional, and preventive domains, the proposed framework aims to avoid labeling minor asymptomatic findings as failure while reducing the risk of overlooking clinically meaningful deficits. Ultimately, it seeks to refocus evaluation on the primary objectives of ACLR: restoring durable knee stability, functional recovery, and patient satisfaction.

In clinical practice, application of this framework requires judgment, as the relevance of symptoms such as pain, instability, or activity limitation may vary among individuals. By contrast, for research purposes, subjective parameters should be operationalized using validated and reproducible thresholds. For example, clinically relevant persistent pain may be defined as ≥ 5/10 on a visual analog scale beyond expected recovery, while functional limitation or patient dissatisfaction may be quantified using PROMs below established PASS cut-offs for instruments such as the IKDC or Lysholm. Importantly, PROMs and PASS thresholds should be interpreted as measures of overall functional outcome and patient satisfaction rather than direct indicators of instability. This approach balances flexibility in clinical interpretation with methodological reproducibility in research settings.

## Conclusions

Failure after ACLR has traditionally been defined using structural endpoints such as graft rupture or revision surgery. However, the available evidence suggests that additional dimensions may be relevant when evaluating outcomes after reconstruction. On the basis of these observations, we propose a conceptual framework that distinguishes between structural failure and functional failure. Structural failure includes graft rupture and pathological laxity, whereas secondary meniscal damage is incorporated within the proposed framework as a potential indicator of persistent biomechanical insufficiency. Functional failure, by contrast, refers to persistent pain, instability-related symptoms, functional limitations, or patient dissatisfaction despite apparent graft integrity.

Although these domains are closely related, they may represent different manifestations of an unsuccessful outcome after ACLR. We therefore propose that considering both structural and functional factors may provide a more comprehensive and patient-centered approach to evaluating reconstruction outcomes. This framework should be viewed as a conceptual model intended to stimulate future discussion, clinical validation studies, and consensus development rather than as a universally accepted definition of ACLR failure. If validated, such an approach could improve consistency in research reporting and better align outcome assessment with the broader objectives of ACLR, including mechanical stability, functional recovery, and joint preservation.

## Data Availability

No datasets were generated or analyzed during the current study.

## Abbreviations

ACL: Anterior cruciate ligament
ACLR: Anterior cruciate ligament reconstruction
ALL: Anterolateral ligament
AP: Anteroposterior
CT: Computed tomography
3D: Three-dimensional
DP-FS: Proton density fat-suppressed
ESSKA: European Society of Sports Traumatology, Knee Surgery and Arthroscopy
IKDC: International Knee Documentation Committee
KOOS: Knee Injury and Osteoarthritis Outcome Score
LEAPs: Lateral extra-articular procedures
MCL: Medial collateral ligament
MRI: Magnetic resonance imaging
PASS: Patient acceptable symptom state
PCL: Posterior cruciate ligament
PROMs: Patient-reported outcome measures
ROM: Range of motion
SANTI: Scientific Anterior Cruciate Ligament Network International
THC: Tetrahydrocannabinol
